# IMPROVING LITERACY OF ALZHEIMER’S DISEASE AND RELATED DISORDERS (ADRD) AMONG OLDER ASIAN AMERICANS IN MICHIGAN

**DOI:** 10.1093/geroni/igae098.4220

**Published:** 2024-12-31

**Authors:** Fei Sun, Kangyi Zhou, Arienne Patano, Daniel Velez-Ortiz, Zhiqi Yi

**Affiliations:** Michigan State University, Haslett, Michigan, United States; Walt Whitman High School, Potomac, Maryland, United States; Michigan State University, East Lansing, Michigan, United States; Michigan State University, East Lansing, Michigan, United States; University of Kansas, Lawrence, Kansas, United States

## Abstract

This study evaluates a community-based participatory intervention designed to enhance ADRD knowledge and reduce ADRD stigma and worry among older Asian Americans in Michigan. Participants were recruited from senior centers and housing, religious congregations, and community Asian service agencies. They engaged in a 2.5-hour program comprising an educational session and a selected culturally tailored activity (e.g., group meals, mindfulness yoga. Pre- and post-intervention surveys focused on ADRD knowledge, worry, and stigma. The intervention was administered to 15 Korean participants (76.4±10.0 years old, 80% female), 82 Chinese participants (82.2±6.6 years old, 60% female), and 32 Indian participants (74.4±7.27 years old, 56% female) delivered in the participants’ native language; the images and examples were also culturally relevant to participants. For the Korean group, ADRD knowledge significantly increased (Hedges’ g =.60, p <.05), and stigma significantly decreased (Hedges’ g =-1.0, p<.01), though changes in worry were not statistically significant. The Chinese participants showed a similar pattern, with an increase in knowledge (Hedges’ g=.32, p<.01) and a decrease in stigma (Hedges’ g=-0.45, p<.01). The Indian community experienced an improvement in ADRD knowledge (Hedges’ g=.50, p<.01) but no significant changes in stigma or worry. Findings underscore the importance of culturally sensitive community-based interventions to address ADRD literacy gaps among older Asian Americans. While knowledge levels increased across all Asian groups, reducing stigma was only effective in the Korean and Chinese communities. Addressing dementia-related worry remains challenging, suggesting the need for more innovative strategies.

